# Evaluation of the updated 2022 lung-GPA in NSCLC adenocarcinoma patients with brain metastases: analysis of prognostic factors in a German clinical cohort

**DOI:** 10.1186/s13014-026-02805-0

**Published:** 2026-02-24

**Authors:** Niklas Mittenbacher, Jens Christian Philippi, Dirk Vordermark, Daniel Medenwald, Jörg Andreas Müller

**Affiliations:** 1 Department of Radiation Oncology, University Hospital Halle (Saale), Ernst-Grube- Str. 40, 06120 Halle (Saale), Germany; 2 University Clinic for Radiation Therapy, University Hospital Magdeburg A. ö. R, Magdeburg, Germany

**Keywords:** NSCLC-GPA, Lung-GPA, Adenocarcinoma, Prognostic score, Brain metastases, PD-L1

## Abstract

**Background:**

Lung cancer is the most frequent source of brain metastases (BMs), with 20-40% of patients with non-small cell lung cancer (NSCLC) developing BMs during the course of disease. The NSCLC-GPA (Graded Prognostic Assessment), developed by Sperduto et al., aims to estimate overall survival (OS) in lung cancer patients with BMs based on multiple prognostic factors. This study aimed to validate the applicability and prognostic relevance of the updated 2022 NSCLC-GPA in a German clinical cohort, with particular attention to programmed death-ligand 1 (PD-L1) expression and treatment-related outcomes.

**Methods:**

We retrospectively analyzed patients with NSCLC and BMs treated between 2020 and 2022 at a German university hospital. GPA scores were calculated using established parameters: age, Karnofsky Performance Status (KPS), number of BMs, presence of extracranial metastases (ECM), EGFR and ALK mutation status, and PD-L1 expression. Due to the low number of non-adenocarcinoma (NAC) cases in our cohort, only patients with adenocarcinoma (AC) were included. Univariate and multivariate Cox regression models, log-rank tests, and Kaplan–Meier curves for illustrative purposes were used to evaluate associations between prognostic factors and OS.

**Results:**

A total of 110 AC patients met the inclusion criteria. Median OS was 10 months (range: 6-11). Patients with GPA scores of 0-1 and 1.5-2 had significantly worse outcomes compared with the reference group (GPA 3.5-4; median OS: 36 months), with median OS times of 3 and 8 months, respectively (HR: 8.34, 95% CI: 2.53-27.5, *p *= 0.0005 and HR: 5.24, 95% CI: 1.584-17.33, *p *= 0.0067). Patients with a GPA of 2.5-3 had a median OS of 22.5 months (HR: 1.6, 95% CI: 0.469-5.45, *p *= 0.453), which was not statistically significant. Older age (≥ 70 years; HR: 1.95, 95% CI: 1.23-3.11, *p *= 0.0047), low KPS (KPS ≤ 70; HR: 3.88, 95% CI: 2.33-6.46, *p* < 0.0001), a higher number of BMs (HR: 1.58, 95% CI: 1.03-2.41, *p* = 0.035), and the presence of ECM (HR: 2.62, 95% CI: 1.66-4.13, *p* < 0.0001) were all significantly associated with decreased OS. In the multivariate analysis, low KPS and ECM remained independently significant prognostic factors. PD-L1 expression showed no significant association with OS.

**Conclusion:**

Despite shorter OS outcomes in our cohort, the 2022 NSCLC-GPA proved to be a valuable prognostic tool, with lower scores consistently associated with poorer outcomes. In addition, older age, low KPS, ECM, and a higher number of BMs were identified as prognostic factors, with low KPS and ECM remaining independent prognostic factors in the multivariate analysis.

**Supplementary Information:**

The online version contains supplementary material available at 10.1186/s13014-026-02805-0.

## Introduction

Lung cancer is one of the most common types of cancer worldwide and remains the leading cause of cancer-related death, including Germany [1–3]. In 2022, approximately 2.4 million people were diagnosed with lung cancer, and 1.8 million died from the disease during the same period [3]. Although lung cancer mortality rates remain high compared to other cancer types, overall mortality has decreased due to improved screening methods [4]. From a global perspective, lung cancer is the most common cancer in men and the second most frequent in women. In Germany, it ranks second in men and third in women [5–8].

Lung cancer is also the most common source of BMs. Among patients with NSCLC, 20-40% develop BMs during the course of their disease [9]. Due to advances in diagnostic and screening methods, as well as improved treatment options [4, 10], the number of diagnosed BMs is expected to increase as patients experience prolonged survival [11].

These patients represent a highly heterogeneous group with varying prognostic factors and therapeutic options [4, 10]. For example, patients with NSCLC and BMs benefit from a combination of initial radiotherapy (RT) and immune checkpoint inhibitor (ICI)-based therapy compared with ICI-based therapy alone, with a hazard ratio (HR) of 0.45 and a confidence interval (CI) of 0.29-0.72 [12]. In addition, surgical resection of BMs represents a safe and effective treatment option [13].

Sperduto et al. identified and weighted key prognostic factors in patients with BMs, leading to the development of a scoring system that has been updated several times in recent years. For NSCLC patients with AC, the score incorporates major determinants of OS, including age, KPS, number of BMs, presence of ECM, and EGFR and ALK mutation status [14].

In 2022, PD-L1 expression was added to the scoring system as an additional significant predictor of OS. In this model, high PD-L1 expression (75-100%) served as the reference category, whereas negative expression (< 1%) was associated with a HR of 1.41 (95% CI: 1.17-1.70, *p* < 0.01).

The GPA groups demonstrated clear survival stratification. Low GPA scores (0-1) were associated with worse survival outcomes compared with higher scores: median survival ranged from 6 months for GPA 0-1 to 15 months for GPA 1.5-2, 30 months for GPA 2.5-3, and 52 months for GPA 3.5-4. This updated scoring system is referred to as the NSCLC-GPA [15].

The NSCLC-GPA is a comprehensive tool designed to estimate survival probabilities in NSCLC patients by incorporating individual risk profiles. In the Sperduto study, which included patient cohorts from different regions such as the USA, Canada, and Japan, median survival ranged from 6 to 52 months [15]. However, these values are cohort-specific and cannot be directly generalized to all NSCLC patients. The primary purpose of the score is to estimate survival probabilities based on individual risk factors and to support personalized treatment strategies for lung cancer patients with BMs.

Therefore, the aim of this study was to evaluate the updated version of the NSCLC-GPA in a real-world clinical setting at a German university hospital, with particular focus on PD-L1 expression as an incorporated prognostic factor. In addition, we also provide an overview of initial treatment strategies.

## Methods and material

Patients were identified retrospectively using the databases of University Hospital Halle. The patient data were anonymized and retrieved from the hospital information system ORBIS (version 03.20.02.01). Information regarding imaging of BMs and their radiation treatment plans were obtained from Centricity PACS by GE Healthcare Integrated IT Solutions Inc. and at Elekta Mosaiq (version 2.84). Initially, all lung cancer patients with BMs treated between January 1, 2020, and December 31, 2022, were considered. After the initial screening process, further analysis focused on NSCLC patients who had developed BMs. Moreover, patients had to have either received their initial BM diagnosis or started their treatment during this period. The subsequent data collection primarily focused on information related to the NSCLC-GPA score [15].

During the study period, only 26 patients with NAC met the required criteria. This number was insufficient to provide statistically robust and reproducible data. The small NAC sample size resulted in insufficient statistical power and an increased risk of type II errors. To ensure robust and reproducible results, the NAC cohort was excluded from further analysis. Therefore, all analyses were based on the AC cohort. Descriptive data from NAC patients are summarized in Table 1.

**Table 1:** Patient characteristics

		Adenocarcinoma (*N* = 110)	Non-Adenocarcinoma (*N* = 26)	Overall (*N* = 136)
Age	< 60	34 (30.9%)	4 (15.4%)	38 (27.9%)
	60–64	24 (21.8%)	4 (15.4%)	28 (20.6%)
	65–69	25 (22.7%)	9 (34.6%)	34 (25.0%)
	70–74	14 (12.7%)	4 (15.4%)	18 (13.2%)
	≥ 75	13 (11.8%)	5 (19.2%)	18 (13.2%)
Sex	Female	43 (39.1%)	7 (26.9%)	50 (36.8%)
	Male	67 (60.9%)	19 (73.1%)	86 (63.2%)
KPS	100	6 (5.5%)	0 (0%)	6 (4.4%)
	90	31 (28.2%)	2 (7.7%)	33 (24.3%)
	80	20 (18.2%)	1 (3.8%)	21 (15.4%)
	70	27 (24.5%)	16 (61.5%)	43 (31.6%)
	≤ 60	26 (23.6%)	7 (26.9%)	33 (24.3%)
ECM	Absent	44 (40.0%)	8 (30.8%)	52 (38.2%)
	Present	66 (60.0%)	18 (69.2%)	84 (61.8%)
Number of BM	1	28 (25.5%)	8 (30.8%)	36 (26.5%)
	2–4	41 (37.3%)	3 (11.5%)	44 (32.4%)
	5–10	35 (31.8%)	13 (50.0%)	48 (35.3%)
	> 10	6 (5.5%)	2 (7.7%)	8 (5.9%)
EGFR	Positive	8 (7.3%)	0 (0%)	8 (5.9%)
	Negative	70 (63.6%)	10 (38.5%)	80 (58.8%)
	Unknown	32 (29.1%)	16 (61.5%)	48 (35.3%)
ALK	Positive	0 (0%)	0 (0%)	0 (0%)
	Negative	75 (68.2%)	11 (42.3%)	86 (63.2%)
	Unknown	35 (31.8%)	15 (57.7%)	50 (36.8%)
PD-L1	Positive	58 (52.7%)	11 (42.3%)	69 (50.7%)
	Negative	43 (39.1%)	11 (42.3%)	54 (39.7%)
	Unknown	9 (8.2%)	4 (15.4%)	13 (9.6%)
GPA groups	0–1	35 (31.8%)	8 (30.8%)	43 (31.6%)
	1.5-2	37 (33.6%)	11 (42.3%)	48 (35.3%)
	2.5-3	32 (29.1%)	6 (23.1%)	38 (27.9%)
	3.5-4	6 (5.5%)	1 (3.8%)	7 (5.1%)
Intracranial progression	Yes	45 (40.9%)	6 (23.1%)	51 (37.5%)
	No	30 (27.3%)	6 (23.1%)	36 (26.5%)
	Missing	35 (31.8%)	14 (53.8%)	49 (36.0%)

OS and intracranial progression-free survival (icPFS) were defined as the primary endpoints. OS was defined as the time from initial diagnosis of BMs to death or last known follow-up, as documented in the local citizen registry. IcPFS was defined as the interval between the date of initial BM diagnosis and the first follow-up imaging demonstrating intracranial progression according to RECIST criteria, or the last imaging without evidence of progression. Some patients were excluded from the icPFS analysis due to death before first follow-up imaging or continuation of treatment at external institutions. These cases are labeled as “missing” in Table 1.

According to the criteria of Sperduto et al., patients with leptomeningeal metastases, patients who did not receive BM-directed treatment, and those treated with best supportive care (BSC) alone were excluded [15].

The NSCLC-GPA score was calculated for each patient (range 0-4) according to the definition by Sperduto et al. (Table 2). All variables used for score calculation were assessed at the time of BM diagnosis. If KPS was not documented at diagnosis, the KPS at the time of first brain radiotherapy was used. EGFR and ALK mutation status as well as PD-L1 expression were typically determined during the initial diagnostic workup of NSCLC. When not reassessed at BM diagnosis, these initial molecular pathology results were used.

**Table 2:** Calculation of the 2022 NSCLC specific lung GPA (adenocarcinoma) based on the model published by Sperduto et al. [15]

Prognostic factor	0	0.5	1	Total score
KPS	≤ 70	80	90–100	0.0–1.0 → GPA group 1
Age	≥ 70	< 70	/	1.5-2.0 → GPA group 2
Number BM	≥ 5	1–4	/	2.5-3.0 → GPA group 3
ECM	Present	/	Absent	3.5-4.0 → GPA group 4
EGFR and ALK	Both negative/unknown	EGFR or ALK positive	/	
PD-L1	Negative/unknown	Positive	/	

Survival curves were estimated using the Kaplan-Meier method to illustrate cumulative survival. Group comparisons were performed using the log-rank test. A p-value < 0.05 was considered statistically significant. To evaluate the prognostic impact of NSCLC-GPA factors, Cox regression analysis were performed. Univariate models were first used to screen potential prognostic variables, followed by multivariate analysis including all significant factors. Statistical analyses were conducted using RStudio (version 2024.04.2 + 764). The R packages used are listed in the references [16–30]. ChatGPT (OpenAI, USA) was used exclusively for language refinement and editorial assistance. All scientific content and interpretations were developed and verified by the authors.

## Results

### Patient characteristics

In the AC subgroup (*n* = 110), 30.9% of patients were younger than 60 years, and the most frequent age group was 65-69 years (22.7%). A male predominance was observed (60.9%). Performance status was moderate to poor in a substantial proportion of patients, with 24.5% presenting with a KPS of 70% and 23.6% with a KPS ≤ 60%. ECM were present in 60.0% of AC cases. Regarding BMs, 25.5% of patients had a single lesion, 37.3% had 2-4 metastases, and 31.8% had more than 5 BMs. EGFR mutations were detected in 7.3% of patients, while 63.6% were EGFR-negative and 29.1% had unknown status. No ALK mutations were detected; 68.2% were ALK-negative and 31.8% had unknown ALK status. Regarding NSCLC-GPA categories, 37 patients (33.6%) were in the 1.5-2 group, 35 (31.8%) in the 0-1 group, and 32 (29.1%) in the 2.5-3 group. PD-L1 expression was positive in 52.7% of cases. Among these patients, 67.2% received PD-L1-targeted immunotherapy (39 of 58 patients). Conversely, 23.6% of patients with negative or unknown PD-L1 status also received immunotherapy, whereas 32.7% of PD-L1-positive patients did not (19 of 58 patients). Intracranial progression occurred in 40.9% of AC patients, while 27.3% showed no evidence of progression; data were missing in 31.8% of cases.

All findings related to initial treatment strategies are summarized in Table 3. Table 1 summarizes patient characteristics by histological subtype (AC vs. NAC) in the overall cohort (*n* = 136). Table 3 details initial BM-directed treatments and the distribution of PD-L1-directed treatments according to molecular status.

**Table 3:** Initial therapy strategies

		Adenocarcinoma (*N* = 110)	Non-Adenocarcinoma (*N* = 26)	Overall (*N* = 136)
Initial therapy brain metastases	Resection	10 (9.1%)	3 (11.5%)	13 (9.6%)
	Resection + Radiotherapy of resection cavity	10 (9.1%)	1 (3.8%)	11 (8.1%)
	Radiotherapy	87 (79.1%)	21 (80.8%)	108 (79.4%)
	Only targeted therapy	3 (2.7%)	1 (3.8%)	4 (2.9%)
Type of cranial radiation	WBRT	14 (12.7%)	7 (26.9%)	21 (15.4%)
	WBRT + simultaneous integrated boost	22 (20.0%)	3 (11.5%)	25 (18.4%)
	WBRT + hippocampal sparing	1 (0.9%)	0 (0%)	1 (0.7%)
	SRT	40 (36.4%)	7 (26.9%)	47 (34.6%)
	Helmet field irradiation	12 (10.9%)	4 (15.4%)	16 (11.8%)
	Radiosurgery	8 (7.3%)	1 (3.8%)	9 (6.6%)
	Patients without radiotherapy	13 (11.8%)	4 (15.4%)	17 (12.5%)
PD-L1 specific therapy	Therapy for positive	39 (35.5%)	9 (34.6%)	48 (35.3%)
	Therapy for negative/ unknown	26 (23.6%)	2 (7.7%)	28 (20.6%)
	No therapy for negative/ unknown	26 (23.6%)	13 (50.0%)	39 (28.7%)
	No therapy for positive	19 (17.3%)	2 (7.7%)	21 (15.4%)

### Survival analyses

Table 4 summarizes OS in AC patients stratified by GPA score. Patients with a GPA of 0-1 (*n* = 35) showed the poorest prognosis, with a median OS of 3 months (range: 2-5 months) and a HR of 8.34 (95% CI: 2.53-27.50, *p* = 0.0005) compared with the reference group (GPA 3.5-4, *n* = 6; median OS: 36 months, range: 34-44 months). Patients with GPA scores of 1.5-2 (*n* = 37) and 2.5-3 (*n* = 32) had median OS values of 8 months (range: 4-11 months) and 22.5 months (range: 15-27.5 months), corresponding to HRs of 5.24 (95% CI: 1.58-17.33, *p* = 0.0067) and 1.60 (95% CI: 0.47-5.45, *p* = 0.453), respectively.

**Fig. 1:**
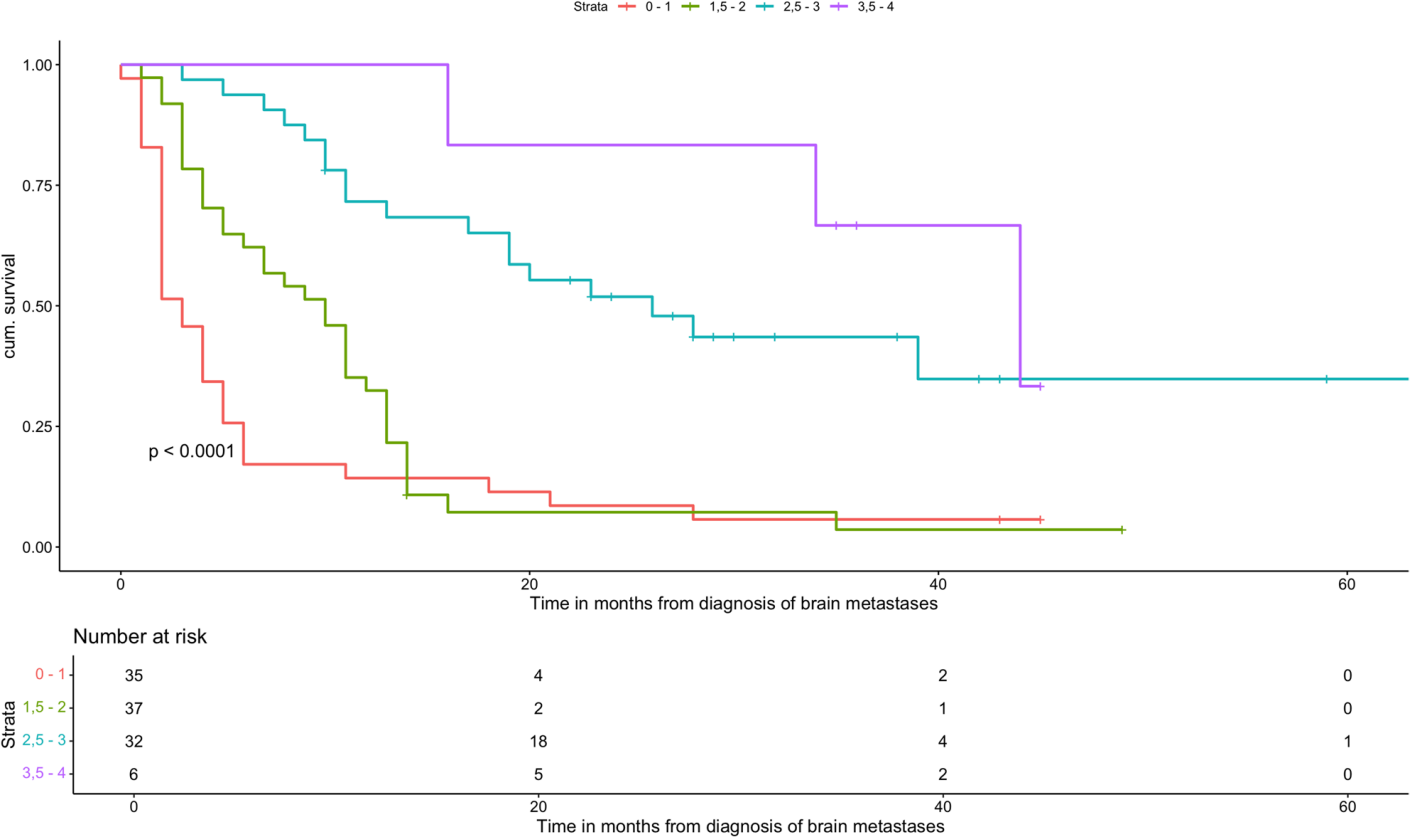
Overall survival based on the GPA of patients with adenocarcinoma

Across GPA groups, increasing GPA scores were associated with progressively improved OS. These results are illustrated in the Kaplan-Meier curves (Fig. 1). Univariate analysis demonstrated a strong inverse relationship between GPA score and mortality risk, with the lowest GPA group (0-1) showing the highest HR.

An exploratory analysis evaluating the predictive value of GPA score and other prognostic factors for icPFS was performed using the Kaplan-Meier method and is provided in the Supplement (S.1). Due to a high proportion of missing icPFS data (31.8%), no further inferential analyses were conducted for this endpoint.

**Table 4:** Comparison of estimated OS from Sperduto et al. and this study

GPA Groups	Sperduto et al.	Observed data
0–1 (*n* = 35)	6 mon. (2–13)	3 mon. (2–5)
1.5–2 (*n* = 37)	15 mon. (5–38)	8 mon. (4–11)
2.5–3 (*n* = 32)	30 mon. (12 - NR)	22.5 mon. (15–27.5)
3.5–4 (*n* = 6)	52 mon. (25–69)	36 mon. (34–44)
Overall (*n* = 110)	17 mon. (6–46)	10 mon. (6–11)

Age ≥ 70 years was associated with significantly worse OS compared to younger patients (HR: 1.95, 95% CI: 1.23-3.11, *p* = 0.0047). Regarding performance status, patients with a KPS of 80 did not show a statistically significant difference in OS compared to fitter patients (KPS 90-100; HR: 1.87, 95% CI: 0.982-3.56, *p* = 0.057), whereas a KPS ≤ 70 was strongly associated with inferior survival (HR: 3.88, 95% CI: 2.33-6.46, *p* < 0.0001). The number of BMs also affected outcomes: patients with five or more BMs had a higher mortality risk than those with fewer lesions (HR: 1.58, 95% CI: 1.03-2.41, *p* = 0.035). In addition, the presence of ECM was significantly associated with reduced OS (HR: 2.62, 95% CI: 1.66-4.13, *p* < 0.0001).

Neither negative nor unknown EGFR mutation status was significantly associated with OS (HR: 1.35, 95% CI: 0.624-2.93, *p* = 0.445). Similarly, negative or unknown PD-L1 expression was not associated with survival differences compared to PD-L1-positive patients (HR: 1.22, 95% CI: 0.801-1.85, *p* = 0.358).

To determine the independent prognostic impact of these variables, a multivariate Cox regression analysis was performed (Table 5). In this model, two factors remained statistically significant. A KPS ≤ 70 was independently associated with reduced OS (adjusted HR: 2.80, 95% CI: 1.630-4.797, *p* = 0.00019), and the presence of ECM remained a significant negative prognostic factor (adjusted HR: 1.77, 95% CI: 1.09-2.885, *p* = 0.021). These results underscore the association of higher performance status and absence of ECM with prolonged OS.

**Fig. 2:**
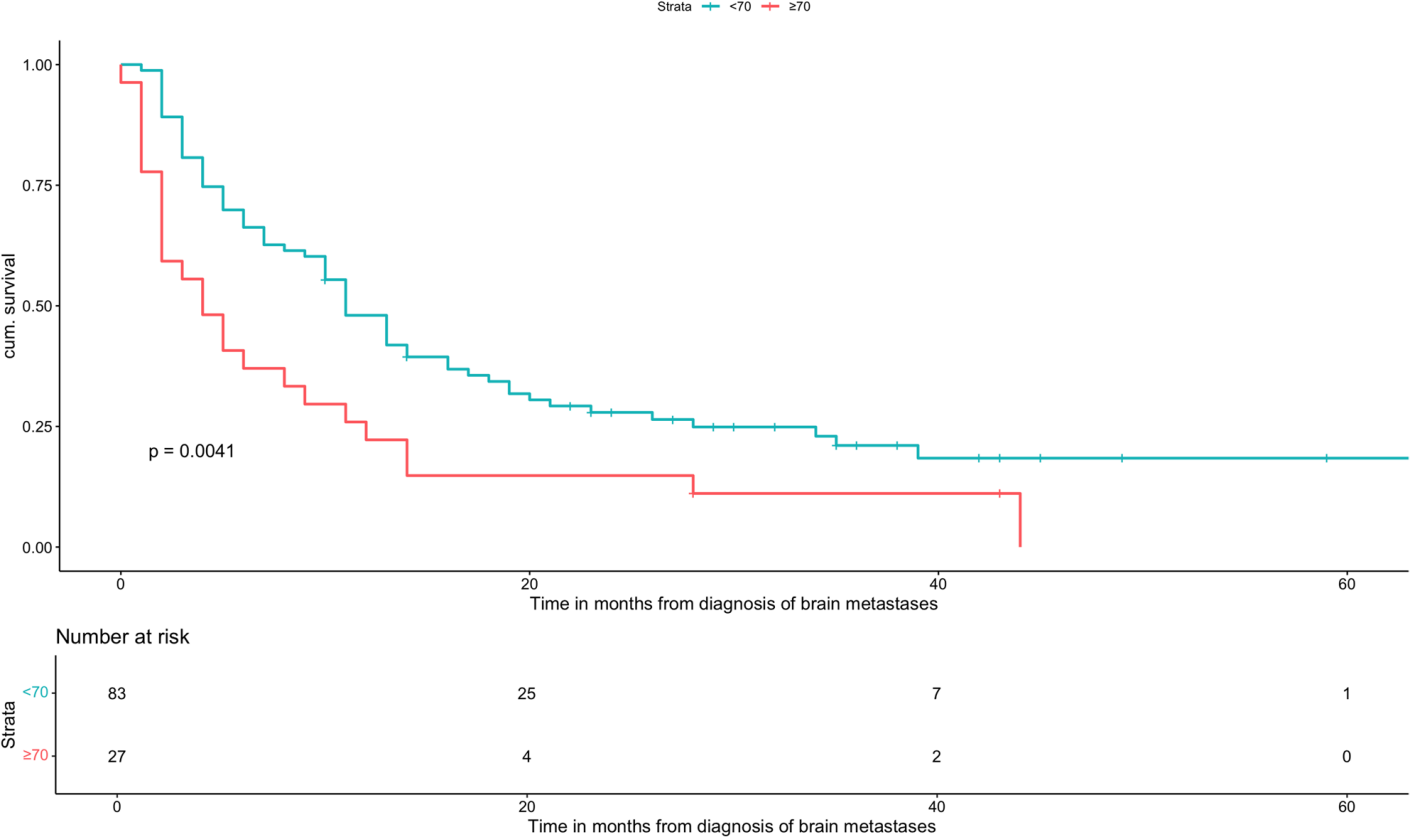
Overall survival based on the age of patients with adenocarcinoma

**Fig. 3:**
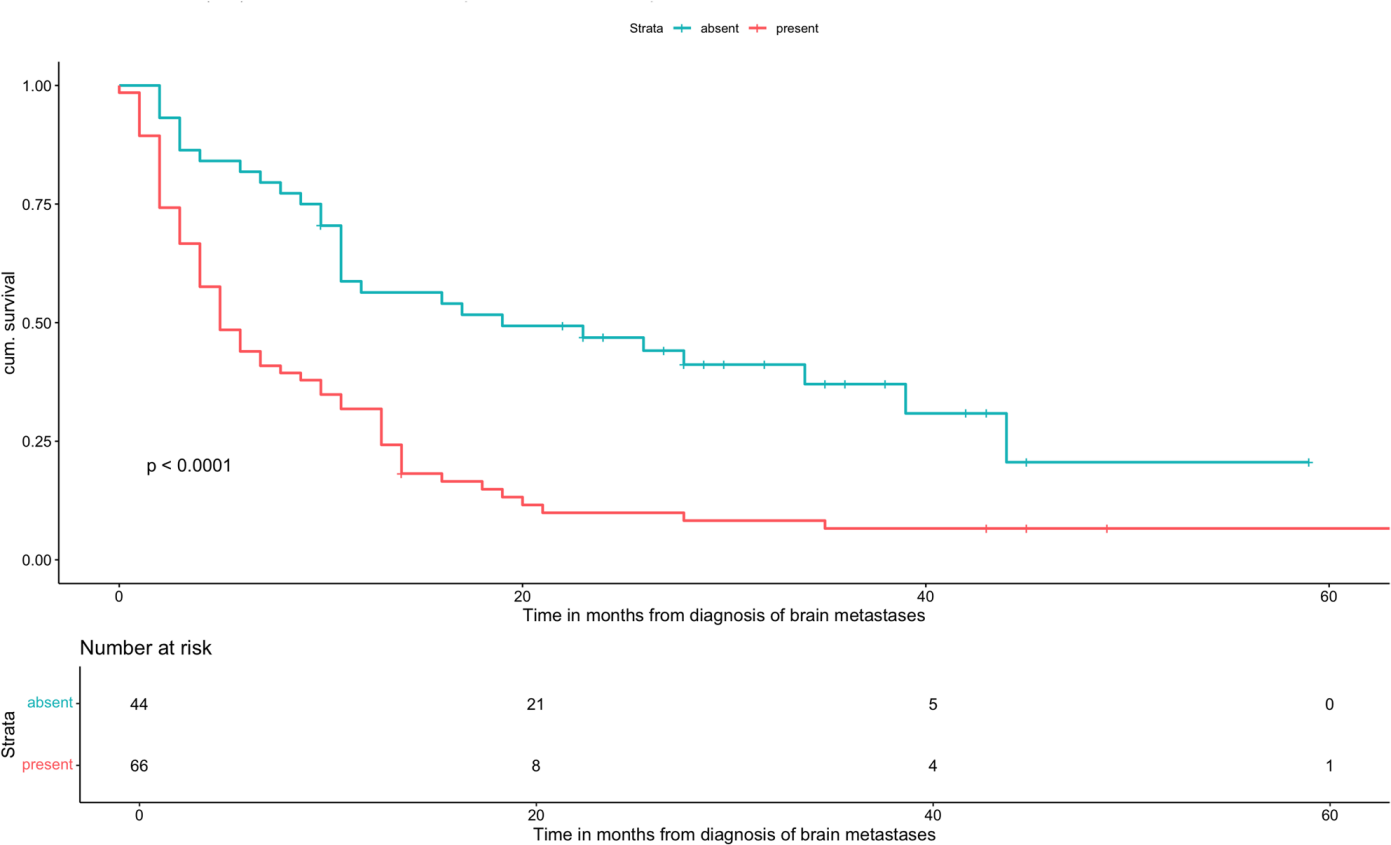
Overall survival based on the absence/ presence of ECM of patients with adenocarcinoma

**Fig. 4:**
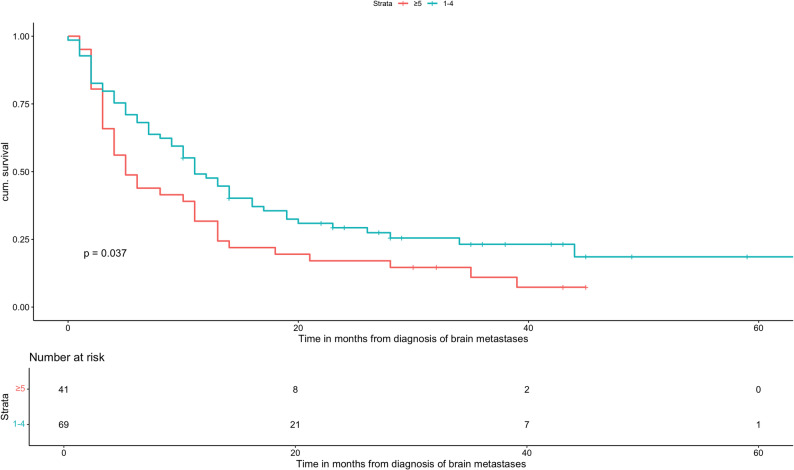
Overall survival based on the number of BMs of patients with adenocarcinoma

**Fig. 5:**
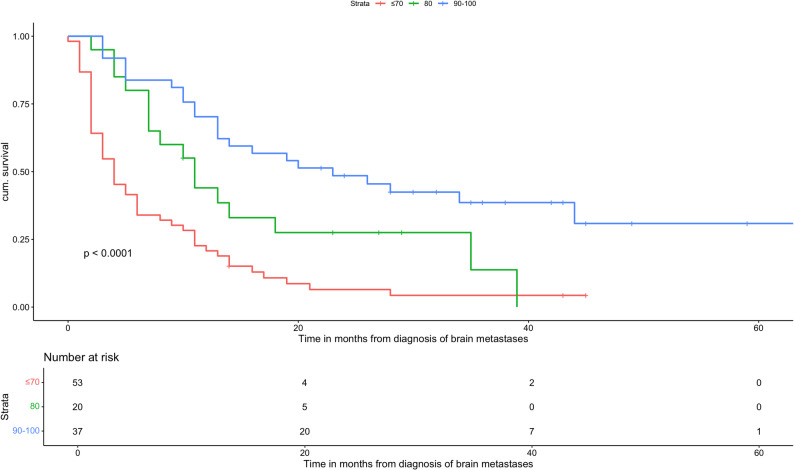
Overall survival based on the KPS of patients with adenocarcinoma

Univariate analysis (Table 5) further demonstrated that younger age, absence of ECM, fewer than five brain metastases, and higher KPS scores were significantly associated with improved OS. Kaplan–Meier curves were generated for all prognostic factors showing significance in univariate analysis (Figs. 2, 3, 4 and 5). PD-L1 expression alone was not significantly associated with OS.

**Fig. 6:**
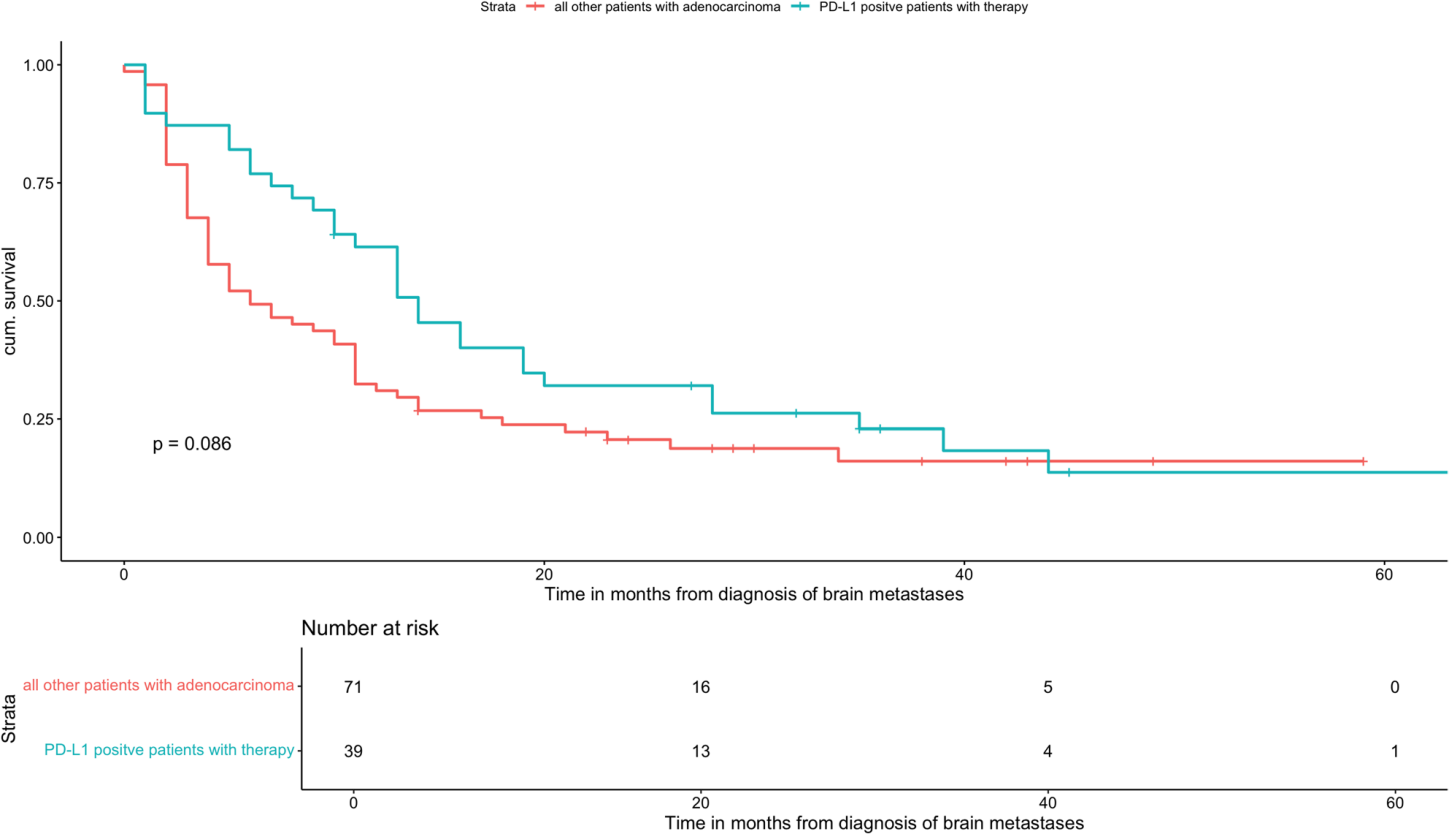
Comparison of overall survival based on received PD-L1 specific therapy in PD-L1 positive patients vs. all other patients with adenocarcinoma (after diagnosis of BMs)

In addition, systemic therapies administered after BM diagnosis were analyzed (Table 3). Notably, more than 32% of PD-L1-positive patients (19 of 58) did not receive PD-L1-targeted therapy. A Kaplan–Meier analysis comparing PD-L1-positive patients who received PD-L1-targeted treatment with all other patients is shown in Fig. 6. The HR for OS in PD-L1-positive patients treated with PD-L1 inhibitors compared with all patients without PD-L1 positivity or PD-L1-directed therapy was 1.45 (HR: 1.477, 95% CI: 0.952-1.85, *p* = 0.081).

**Table 5:** Cox regression analysis for overall survival

		Univariate analysis		Multivariate analysis	
		HR (95% CI)	p value	HR (95%CI)	p value
Age	< 70	1.0 (reference)		1.0 (reference)	
	≥ 70	1.95 (1.23–3.11)	0.0047	1.48 (0.896–2.459)	0.125
KPS	90–100	1.0 (reference)		1.0 (reference)	
	80	1.87 (0.982–3.56)	0.057	1.68 (0.871–3.228)	0.122
	≤ 70	3.88 (2.33–6.46)	< 0.0001	2.80 (1.630–4.797)	0.00019
Number of BM	1–4	1.0 (reference)		1.0 (reference)	
	≥ 5	1.58 (1.03–2.41)	0.035	1.40 (0.892–2.195)	0.144
ECM	Absent	1.0 (reference)		1.0 (reference)	
	Present	2.62 (1.66–4.13)	< 0.0001	1.77 (1.09–2.885)	0.021
EGFR	Positive	1.0 (reference)			
	Negative/unknown	1.35 (0.624–2.93)	0.445		
PD-L1	Positive	1.0 (reference)			
	Negative/unknown	1.22 (0.801–1.85)	0.358		
PD-L1 therapy	Positve with therapy	1.0 (reference)			
	All other patients	1.477 (0.952–2.291)	0.081		
GPA	3.5–4	1.0 (reference)			
	2.5–3	1.6 (0.469–5.45)	0.453		
	1.5–2	5.24 (1.584–17.33)	0.0067		
	0–1	8.34 (2.53–27.5)	0.0005		

## Discussion

The aim of this study was to evaluate the prognostic value of the NSCLC-GPA in a real-world clinical setting and to assess whether this prognostic score can be reliably applied to a different geographic region and healthcare environment.

The cohort analyzed by Sperduto et al. was recruited from the USA, Canada, and Japan [15]. As demonstrated in our German cohort, the prognostic performance of the NSCLC-GPA remained highly significant, with a p-value < 0.0001 based on Kaplan-Meier analysis (Fig. 1). In addition, univariate Cox regression revealed significant differences in median OS across GPA groups (Table 5). Overall, higher GPA scores were consistently associated with improved OS, and this trend was observed across all four GPA categories.

However, compared with the results reported by Sperduto et al., median OS values in our cohort were lower across all GPA groups. This discrepancy may, at least in part, be explained by the smaller sample size of our study (110 patients versus 2856 patients). Furthermore, the Sperduto study was based on a large, international, multicenter dataset including 20 institutions, which may limit direct comparability of absolute survival outcomes [15].

Radiotherapy strategies were broadly comparable between the two cohorts. In our study, stereotactic radiotherapy (SRT) was the most frequently applied modality (36%), consistent with the Sperduto cohort, where SRT accounted for 45% of treatments. Whole-brain radiotherapy (WBRT), either alone or in combination with other radiation techniques, represented the second most common approach in both studies, with 33.6% of patients receiving WBRT in our cohort compared with 24% in the Sperduto cohort. Surgical resection followed by radiotherapy was less frequently performed in our cohort (9.1%) than in the Sperduto cohort (15.2%).

Another important factor potentially contributing to the lower OS observed in our study is the distribution of performance status. In the Sperduto cohort, a larger proportion of patients exhibited higher KPS scores. Specifically, 42% of patients had a KPS of 90-100 compared with 33.7% in our cohort, while 28% had a KPS of 80 versus 18.2% in our study. Conversely, nearly half of our patients (48.1%) had a KPS of 70 or lower, compared with only 27% in the Sperduto cohort. Similar findings have been reported in other real-world studies with less fit patient populations. For example, Schröder et al. reported that only 15.3% of patients had a KPS of 90-100, while 36.5% had a KPS of 70 or lower, accompanied by a reduced median OS of 15 months compared with 17 months in the Sperduto cohort [31].

Sex distribution may also have influenced survival outcomes. In our cohort, 60.9% of patients were male, whereas the Sperduto cohort included a higher proportion of female patients (54%) [15]. Several studies have demonstrated a statistically significant survival advantage for female patients with NSCLC [32, 33]. Notably, female sex was also identified as a significant prognostic factor in the 2022 update of the NSCLC-GPA, although this was the first time the effect had been observed [15].

Comparable European studies have likewise reported lower OS values in certain GPA groups. Schröder et al. observed a median OS of 15 months, compared with 17 months in the Sperduto cohort [31]. Similarly, Crouzen et al. reported lower median OS values for GPA groups 2, 3, and 4 compared with those described by Sperduto et al. [34].

In our cohort, the prognostic impact of EGFR mutation status was only partially evident. Univariate analysis did not demonstrate a statistically significant association with OS, in contrast to findings reported by Sperduto et al. This discrepancy may be attributable to the lower proportion of EGFR-positive patients in our study (7.3%) compared with other cohorts, including Schröder et al. (10%), Crouzen et al. (13.9%), and Sperduto et al. (26%) [15, 31, 34]. In addition, a relatively high proportion of patients had unknown EGFR status (29.1%), limiting the ability to determine whether these patients may have benefited from targeted therapies. Together with the low absolute number of EGFR-positive cases, these factors restrict the statistical power of our analysis. Consistent with our findings, Crouzen et al. also did not observe a significant association between EGFR status and survival [34]. Analysis of ALK mutation status was not feasible due to the absence of ALK-positive cases.

Beyond validating the overall prognostic performance of the NSCLC-GPA, a key objective of this study was to assess the clinical relevance of PD-L1 expression, which was incorporated into the score in 2022. In our initial analyses, PD-L1 positivity was not significantly associated with OS compared with negative or unknown PD-L1 status (*p* = 0.358). PD-L1-positive patients accounted for 52.7% of our cohort, exceeding the proportions reported by Sperduto et al. (38%) and Crouzen et al. (36%) [15, 34].

Given the high data quality and relatively low proportion of missing PD-L1 data, additional exploratory analyses were performed. Stratification according to systemic therapy revealed that more than 32% of PD-L1-positive patients did not receive PD-L1-targeted treatment. This observation suggests that PD-L1 expression alone may have limited prognostic value in the absence of corresponding immunotherapy.

Accordingly, we conducted a Kaplan-Meier analysis comparing PD-L1-positive patients who received PD-L1-targeted therapy with all other patients. This analysis indicated a trend toward improved survival for PD-L1-positive patients treated with PD-L1 inhibitors, although statistical significance was not reached (*p* = 0.086) (Fig. 6). This finding is supported by univariate Cox regression, which demonstrated similar effect estimates (HR 1.477, 95% CI: 0.952-2.291, *p* = 0.081) (Table 5).

This analysis indicated that PD-L1 expression alone was not associated with a survival benefit in our cohort; rather, a potential survival advantage emerged only when PD-L1 expression was combined with PD-L1-targeted therapy. Isolated PD-L1 expression does not confer a survival advantage and may indicate a poorer prognosis compared to PD-L1-negative status. Through its binding to the PD-1 receptor on T-cells, PD-L1 suppresses T-cell activity and induces apoptosis, thereby weakening immune defenses against the tumor. This mechanism promotes tumor growth and progression [35]. Accordingly, PD-L1 expression seems to translate into improved clinical outcomes primarily when accompanied by PD-L1-directed immunotherapy. This observation suggests that PD-L1 expression levels above 1% may have prognostic relevance in the context of appropriate systemic treatment, as previously reported in the literature [35, 36]. In our cohort, this effect could be demonstrated to a limited extent.

Several limitations of this study should be acknowledged. Its retrospective design and relatively small sample size, particularly when compared with the Sperduto trial, restrict the generalizability of the findings. Furthermore, the use of routine clinical data and non-standardized documentation - especially regarding prognostic variables - limited consistent comparability across patients. Follow-up was challenging in a substantial proportion of cases, as many patients transitioned to best supportive care (BSC), often resulting in discontinuation of systematic follow-up. In addition, the NSCLC-GPA does not account for non-oncological comorbidities, which can also impact outcomes and influence personalized treatment decisions [37]. Patients receiving initial BSC were excluded in accordance with the predefined criteria of the Sperduto trial [15]. Karnofsky Performance Status (KPS) emerged as one of the most influential prognostic factors in our analysis. A KPS ≤70 showed a highly significant association with reduced OS (*p* < 0.0001), indicating a strong prognostic impact in our cohort and underscoring its clinical relevance. However, it should be noted that KPS is a subjective measure and may vary between evaluators, despite standardized definitions for score assignment based on patient condition [38].

Including the composite GPA score alongside its individual components (such as age, KPS, and number of BMs) in multivariate models introduces multicollinearity due to the strong correlations between these variables. This can lead to unstable HR estimates, inflated standard errors, and unreliable CI and p-values. Moreover, such models complicate interpretation, as it becomes unclear whether observed effects arise from the GPA score itself or from its constituent factors. For these methodological reasons, multivariate analysis including the GPA score was deliberately not performed, allowing a clearer assessment of the independent prognostic effects of individual variables.

## Conclusion

In the univariate analysis, younger age, higher KPS, absence of ECM, and fewer than five BMs were significantly associated with improved OS. Higher GPA scores correlated with better survival: the lowest GPA group had the poorest prognosis, while the highest GPA group showed the most favorable outcome. In multivariate analysis, only KPS ≤ 70 and the presence of ECM remained independently significant, highlighting their strong prognostic relevance. Despite the study’s limitations and deviations from the original Sperduto cohort, the NSCLC-GPA remains a valuable tool for estimating OS and may support personalized treatment strategies. Future studies should aim to refine the NSCLC-GPA by integrating additional prognostic markers and to further evaluate its role in clinical decision-making.

## Electronic Supplementary Material

Below is the link to the electronic supplementary material.

Supplementary Material 1

## Data Availability

No datasets were generated or analysed during the current study.

## Abbreviations

NSCLC: Non-small cell lung cancer
OS: Overall survival
GPA: Graded Prognostic Assessment
KPS: Karnofsky performance status
EGFR: Epidermal growth factor
ALK: Anaplastic lymphom kinase
PD-L1: Programmed death-ligand 1
HR: Hazard ratio
CI: Confidence intervall
ECM: Extracranial metastases
BM: Brain metastases
icPFS: Intracranial progression-free survival
BSC: Best supportive care
WBRT: Whole brain radiotherapy
SRT: Stereotactic radiotherapy
NAC: Non-Adenocarcinoma
AC: Adenocarcinoma
RT: Radiation therapy
NR: Not reached
siB: Simultaneous integrated boost
ICI: Immun checkpoint inhibitor

## References

[1] Sung H, Ferlay J, Siegel RL, Laversanne M, Soerjomataram I, Jemal A, Bray F. Global cancer statistics 2020: GLOBOCAN estimates of incidence and mortality worldwide for 36 cancers in 185 countries. CA Cancer J Clin. 2021;71:209–49. 10.3322/caac.21660. 33538338 10.3322/caac.21660

[2] Statistisches Bundesamt. Die 10 häufigsten Todesfälle durch Krebs. 2023. https://www.destatis.de/DE/Themen/Gesellschaft-Umwelt/Gesundheit/Todesursachen/Tabellen/sterbefaelle-krebs-insgesamt.html. Accessed 16 Oct 2024.

[3] 2024. Global Cancer Observatory: Cancer Today. Lyon, France: International Agency for Research on Cancer. Available from: https://gco.iarc.who.int/today, accessed [23.10.2024]. (Ferlay J, Ervik M, Lam F, Laversanne M, Colombet M, Mery L, Piñeros M, Znaor A, Soerjomataram I, Bray F). https://gco.iarc.who.int/media/globocan/factsheets/cancers/15-trachea-bronchus-and-lung-fact-sheet.pdf.

[4] Alexander M, Kim SY, Cheng H, Update. 2020: Management of non-small cell lung cancer. Lung. 2020;198:897–907. 10.1007/s00408-020-00407-5. 10.1007/s00408-020-00407-5 PMC7656891 33175991

[5] Langer T. S3-Leitlinie Prävention, Diagnostik, Therapie und Nachsorge des Lungenkarzinoms: Version 3.0 - März 2024 AWMF-Registernummer: 020-007OL, pp. 35–41.

[6] RKI. RKI – Krebs in Deutschland – 2019/2020: p. 16.

[7] Thandra KC, Barsouk A, Saginala K, Aluru JS, Barsouk A. Epidemiology of lung cancer. Contemp Oncol (Pozn). 2021;25(1):45–52. 10.5114/wo.2021.103829. Epub 2021 Feb 23. PMID: 33911981; PMCID: PMC8063897. 33911981 10.5114/wo.2021.103829 PMC8063897

[8] 2024. Global Cancer Observatory: Cancer Today. Lyon, France: International Agency for Research on Cancer. Available from: https://gco.iarc.who.int/today, accessed [DD Month YYYY]. (Ferlay J, Ervik M, Lam F, Laversanne M, Colombet M, Mery L, Piñeros M, Znaor A, Soerjomataram I, Bray F). https://gco.iarc.who.int/media/globocan/factsheets/populations/900-world-fact-sheet.pdf. Accessed 24 Oct 2024.

[9] Villano JL, Durbin EB, Normandeau C, Thakkar JP, Moirangthem V, Davis FG. Incidence of brain metastasis at initial presentation of lung cancer. Neuro Oncol. 2015;17:122–8. 10.1093/neuonc/nou099. 24891450 10.1093/neuonc/nou099 PMC4483041

[10] Liu S-YM, Zheng M-M, Pan Y, Liu S-Y, Li Y, Wu Y-L. Emerging evidence and treatment paradigm of non-small cell lung cancer. J Hematol Oncol. 2023. 10.1186/s13045-023-01436-2. 37069698 10.1186/s13045-023-01436-2 PMC10108547

[11] Liu Q, Tong X, Wang J. Management of brain metastases: history and the present. Chin Neurosurg Jl. 2019. 10.1186/s41016-018-0149-0. 10.1186/s41016-018-0149-0 PMC7398203 32922901

[12] Tozuka T, Minegishi Y, Yamaguchi O, Watanabe K, Toi Y, Saito R, et al. Immunotherapy with radiotherapy for brain metastases in patients with NSCLC: NEJ060. JTO Clin Res Rep. 2024;5:100655. 10.1016/j.jtocrr.2024.100655. 38706978 10.1016/j.jtocrr.2024.100655 PMC11069015

[13] D’Andrea G, Palombi L, Minniti G, Pesce A, Marchetti P. Brain metastases: surgical treatment and overall survival. World Neurosurg. 2017;97:169–77. 10.1016/j.wneu.2016.09.054. 27667577 10.1016/j.wneu.2016.09.054

[14] Sperduto PW, Yang TJ, Beal K, Pan H, Brown PD, Bangdiwala A, et al. Estimating survival in patients with lung cancer and brain metastases: an update of the graded prognostic assessment for lung cancer using molecular markers (Lung-molGPA). JAMA Oncol. 2017;3:827–31. 10.1001/jamaoncol.2016.3834. 27892978 10.1001/jamaoncol.2016.3834 PMC5824323

[15] Sperduto PW, De B, Li J, Carpenter D, Kirkpatrick J, Milligan M, et al. Graded prognostic assessment (GPA) for patients with lung cancer and brain metastases: initial report of the small cell lung cancer GPA and update of the Non-Small cell lung cancer GPA including the effect of programmed death ligand 1 and other prognostic factors. Int J Radiat Oncol Biol Phys. 2022;114:60–74. 10.1016/j.ijrobp.2022.03.020. 35331827 10.1016/j.ijrobp.2022.03.020 PMC9378572

[16] 2021. Blemings dplyr, A., Allen C.). https://dplyr.tidyverse.org. Accessed 23 Oct 2024.

[17] 2021. gtsummary: Presentation of summary statistics and results from statistical models (2.0.0). R Foundation for Statistical Computing. (DuGoff, E., Schuler, M., & D’Agostino, M.). https://gtsummary.rstudio.com. Accessed 24 Oct 2024.

[18] 2021. Grolemund lubridate, G., Wickham H.). https://lubridate.tidyverse.org. Accessed 24 Oct 2024.

[19] 2021. survival: Survival analysis (3.7-0). R Foundation for Statistical Computing. (Therneau TM.). https://cran.r-project.org/web/packages/survival/index.html. Accessed 24 Oct 2024.

[20] 2021. survminer: Drawing survival curves using ggplot2 (0.4.9). R Foundation for Statistical Computing. (Kassambara, A., & Kosinski, M.). https://cran.r-project.org/web/packages/survminer/index.html. Accessed 24 Oct 2024.

[21] 2021. tidyverse: Easily install and load the ‘tidyverse’ (2.0.0). R Foundation for Statistical Computing. (Wickham H.). https://tidyverse.tidyverse.org. Accessed 24 Oct 2024.

[22] 2021. Gohel readxl. V.). https://cran.r-project.org/web/packages/readxl/index.html. Accessed 24 Oct 2024.

[23] 2021. labelled: Label data frames and vectors (2.13.0). R Foundation for Statistical Computing. (Lepage J.). https://cran.r-project.org/web/packages/labelled/index.html. Accessed 24 Oct 2024.

[24] 2021. openxlsx: Read, write and edit Excel files (4.2.6.1). R Foundation for Statistical Computing. (Yoon J, Kim S.). https://cran.r-project.org/web/packages/openxlsx/index.html. Accessed 24 Oct 2024.

[25] 2021. Table 1: Create Table 1 for clinical papers (1.4.3). R Foundation for Statistical Computing. (Hoffman M, Hwang J.). https://cran.r-project.org/web/packages/table1/index.html. Accessed 24 Oct 2024.

[26] 2021. boot: Bootstrap R (1.3–30). R Foundation for Statistical Computing. (Efron B, Tibshirani RJ.). https://cran.r-project.org/web/packages/boot/index.html. Accessed 24 Oct 2024.

[27] 2021. flextable: Create and format tables in Word and PowerPoint documents (0.9.6). R Foundation for Statistical Computing. (Graham, N.). https://cran.r-project.org/web/packages/flextable/index.html. Accessed 24 Oct 2024.

[28] 2021. officer: Interact with Microsoft Word and PowerPoint documents (0.6.7). R Foundation for Statistical Computing. (Xie Y.). https://cran.r-project.org/web/packages/officer/index.html. Accessed 24 Oct 2024.

[29] 2021. broom: Convert statistical analysis objects into tidy tibbles (1.0.7). R Foundation for Statistical Computing. (Robinson D, Hayes A.). Accessed 24 Oct 2024.

[30] 2021. MatchIt. Nonparametric Preprocessing for Parametric Causal Inference (4.5.5). R Foundation for Statistical Computing. (Ho, D. E., Imai, K., King, G., & Stuart, E. A.). https://cran.r-project.org/web/packages/MatchIt/index.html.

[31] Schröder C, Windisch P, Lütscher J, Zwahlen DR, Förster R. Validation and discussion of clinical practicability of the 2022 graded prognostic assessment for NSCLC adenocarcinoma patients with brain metastases in a routine clinical cohort. 10.3389/fonc.2023.1042548 PMC10067866 37020868

[32] Hsu L-H, Chu N-M, Liu C-C, Tsai SYC, You D-L, Ko J-S, et al. Sex-associated differences in non-small cell lung cancer in the new era: is gender an independent prognostic factor? Lung Cancer. 2009;66:262–7. 10.1016/j.lungcan.2009.01.020. 19299032 10.1016/j.lungcan.2009.01.020

[33] Wakelee HA, Wang W, Schiller JH, Langer CJ, Sandler AB, Belani CP, Johnson DH. Survival differences by sex for patients with advanced Non-small cell lung cancer on Eastern cooperative oncology group trial 1594. J Thorac Oncol. 2006;1:441–6. 10.1016/S1556-0864(15)31609-9. 17409897

[34] Crouzen JA, Mast ME, Hakstege M, Broekman MLD, Baladi C, Mertens BJA, et al. External validation of the lung-molGPA to predict survival in patients treated with stereotactic radiotherapy for brain metastases of non-small cell lung cancer. Radiother Oncol. 2024;198:110405. 10.1016/j.radonc.2024.110405. 38925263 10.1016/j.radonc.2024.110405

[35] PD-1/PD-L1 pathway. Current researches in cancer, Yanyan Han1, Dandan Liu2, Lianhong Li1. Am J Cancer Res. 2020;10(3):727–42. ISSN:2156–6976/ajcr0108072. 32266087 PMC7136921

[36] Yi M, Zheng X, Niu M, Zhu S, Ge H, Wu K. Combination strategies with PD-1/PD-L1 blockade: current advances and future directions. Mol Cancer. 2022;21:28. 10.1186/s12943-021-01489-2. 35062949 10.1186/s12943-021-01489-2 PMC8780712

[37] Ngeow J, Leong SS, Gao F, Toh CK, Lim WT, Tan EH, Poon D. Impact of comorbidities on clinical outcomes in non-small cell lung cancer patients who are elderly and/or have poor performance status. Crit Rev Oncol Hematol 2010 Oct, 1:53–60. 10.1016/j.critrevonc.2009.10.005. Epub 2009 Nov 24. PMID: 19939700. (76). 10.1016/j.critrevonc.2009.10.005 19939700

[38] Péus D, Newcomb N, Hofer S. Appraisal of the Karnofsky performance status and proposal of a simple algorithmic system for its evaluation. BMC Med Inf Decis Mak. 13, 72. 1472-6947-13–72. 10.1186/1472-6947-13-72 PMC3722041 23870327

