# Supplementary figures and images for: Evaluation of the updated 2022 lung-GPA in NSCLC adenocarcinoma patients with brain metastases: analysis of prognostic factors in a German clinical cohort
Source: Radiat Oncol. 2026 Feb 24;21:40. doi: 10.1186/s13014-026-02805-0 (PMC12964932; doi:10.1186/s13014-026-02805-0)

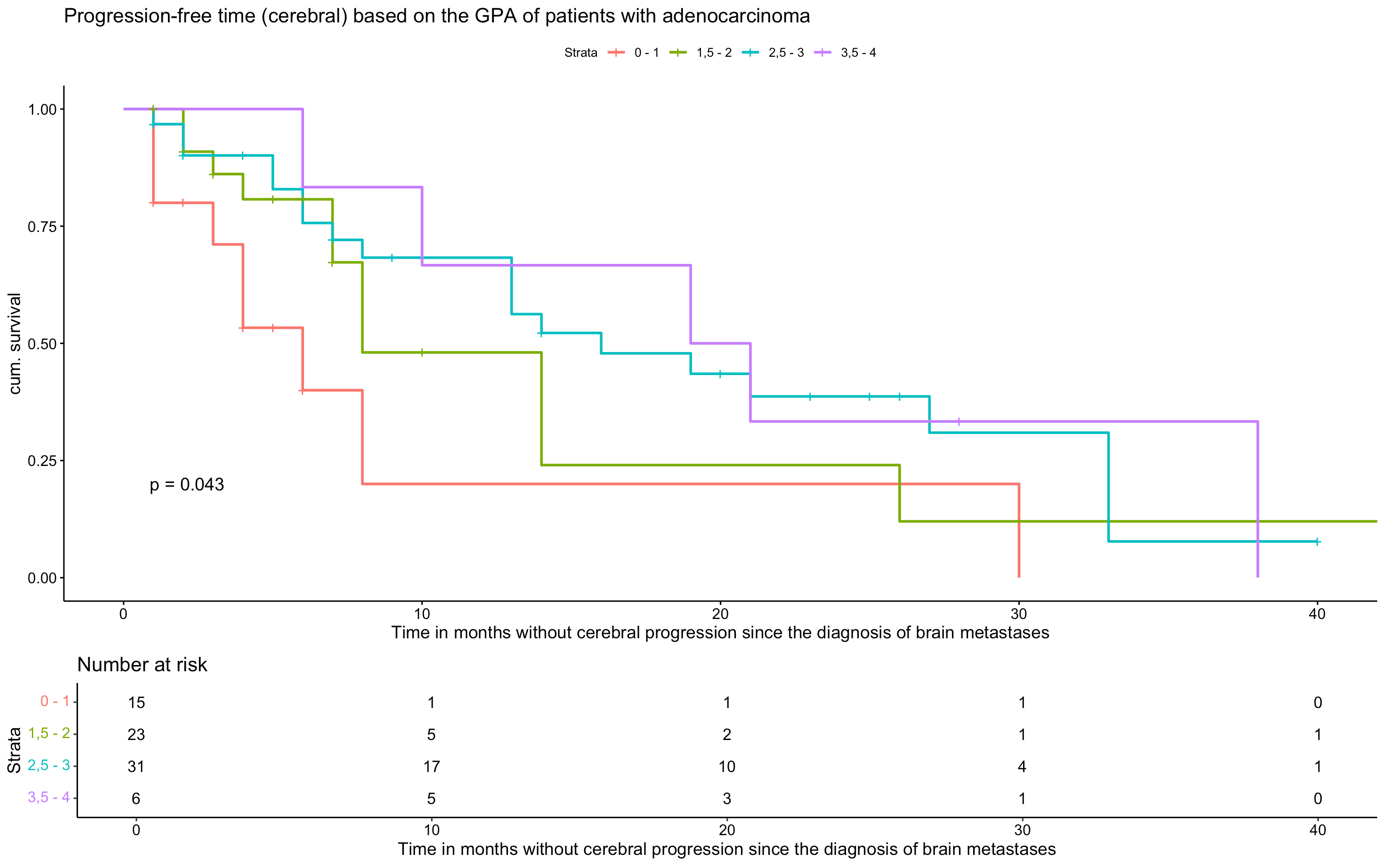


**S. 1**

Supplement: Supplementary file 1 — Supplementary Material 1 [file 13014_2026_2805_MOESM1_ESM.docx]
